# A pilot safety and tolerability study of scanning ultrasound as a neuromodulation therapy in Alzheimer’s disease

**DOI:** 10.1093/braincomms/fcaf445

**Published:** 2025-12-15

**Authors:** Peter J Nestor, Matthew Pelekanos, Gerhard Leinenga, Jae Song, Wendy Lee, Gina Richter-Stretton, Caitlin McElligott, Amir Fazlollahi, Jason B Mattingley, Anthony Harris, Henry Beale, Jennie Roberts, Rachel de las Heras, Jürgen Götz

**Affiliations:** Clem Jones Centre for Ageing Dementia Research, Queensland Brain Institute, The University of Queensland, Brisbane 4072, Australia; Cognitive Health Program, Mater Research Institute and Department of Neurology, Mater Adult Hospital, Mater Misericordiae Limited, South Brisbane 4101, Australia; Queensland Brain Institute, The University of Queensland, Brisbane 4072, Australia; NHMRC Centre of Research Excellence in Mechanisms in NeuroDegeneration—Alzheimer's Disease, The University of Queensland, Brisbane 4072, Australia; Clem Jones Centre for Ageing Dementia Research, Queensland Brain Institute, The University of Queensland, Brisbane 4072, Australia; Clem Jones Centre for Ageing Dementia Research, Queensland Brain Institute, The University of Queensland, Brisbane 4072, Australia; Clem Jones Centre for Ageing Dementia Research, Queensland Brain Institute, The University of Queensland, Brisbane 4072, Australia; Clem Jones Centre for Ageing Dementia Research, Queensland Brain Institute, The University of Queensland, Brisbane 4072, Australia; Clem Jones Centre for Ageing Dementia Research, Queensland Brain Institute, The University of Queensland, Brisbane 4072, Australia; Cognitive Health Program, Mater Research Institute and Department of Neurology, Mater Adult Hospital, Mater Misericordiae Limited, South Brisbane 4101, Australia; Clem Jones Centre for Ageing Dementia Research, Queensland Brain Institute, The University of Queensland, Brisbane 4072, Australia; Queensland Brain Institute, The University of Queensland, Brisbane 4072, Australia; NHMRC Centre of Research Excellence in Mechanisms in NeuroDegeneration—Alzheimer's Disease, The University of Queensland, Brisbane 4072, Australia; School of Psychology, The University of Queensland, Brisbane 4072, Australia; Queensland Brain Institute, The University of Queensland, Brisbane 4072, Australia; School of Psychology, The University of Queensland, Brisbane 4072, Australia; Queensland Brain Institute, The University of Queensland, Brisbane 4072, Australia; Radiology Department, Royal Brisbane and Women’s Hospital, Brisbane 4006, Australia; Clem Jones Centre for Ageing Dementia Research, Queensland Brain Institute, The University of Queensland, Brisbane 4072, Australia; Clem Jones Centre for Ageing Dementia Research, Queensland Brain Institute, The University of Queensland, Brisbane 4072, Australia; NHMRC Centre of Research Excellence in Mechanisms in NeuroDegeneration—Alzheimer's Disease, The University of Queensland, Brisbane 4072, Australia

**Keywords:** Alzheimer’s disease (AD), EEG recording, low-intensity ultrasound, MRI, neuropsychiatric inventory (NPI) test

## Abstract

Clearing amyloid-β pathology in Alzheimer’s disease (AD) has been considered a prerequisite for restoring cognitive functions. Intriguingly, by application of a modality of scanning ultrasound (SUS) to mice that does not remove amyloid-β, we previously achieved significant cognitive improvements. This prompted us to explore SUS as a non-invasive brain stimulation strategy in an open-label safety trial in AD. We conducted a human pilot study in 12 participants with AD with the primary objective of determining feasibility, safety and tolerability. Exploratory secondary end-points were cognitive and behavioural measures, resting-state EEG and functional MRI. A portable device termed UltraThera^Pilot^ was built under medical device standard guidelines, integrating a Brainsight image-guided neuronavigation system. A single-element 286-kHz transducer was programmed to deliver non-derated ultrasound doses of 2.6, 1.95 or 1.3 MPa. With four treatment sessions spaced fortnightly, four participants received 30 sonications per session (precuneus, ∼30 cm^3^ brain tissue) and the remaining 8 received 100 sonications per session (bilateral precuneus and temporo-parietal association cortex, ∼100 cm^3^). Safety monitoring, EEG, MRI, cognitive and neuropsychiatric evaluations were performed. The treatment was fast, safe and well-tolerated at the 1.95 MPa dose. MRI showed no changes, whereas changes were observed in aperiodic EEG content. Cognitive performance did not change but statistically significant improvements in behavioural and psychological symptoms were found using the Neuropsychiatric Inventory test. In conclusion, this SUS safety trial met its primary and secondary end-points in biomarker-confirmed mild-to-moderate AD. It informs our future work in an upcoming efficacy trial in an AD population.

## Teaser

Nestor et al. deployed a portable, handheld neuronavigation-guided low-intensity ultrasound system in a single-arm safety and feasibility study involving 12 Alzheimer’s participants treated 4× with 30–100 sonications at a 1.95 MPa dose. The primary objectives were met. Changes were observed in aperiodic EEG content along with improvements in the Neuropsychiatric Inventory test.

## Graphical Abstract

**Graphical Abstract:**
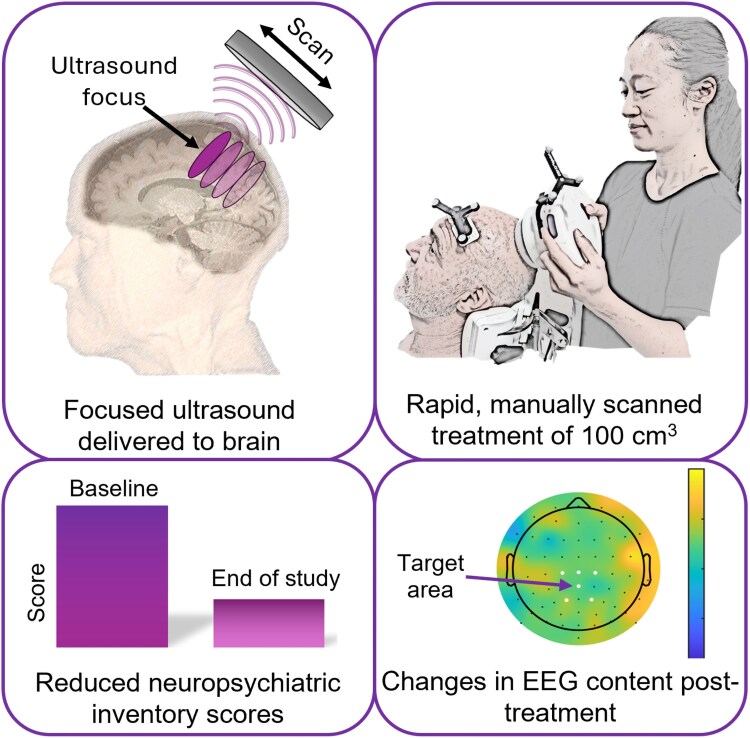

## Introduction

Alzheimer’s disease (AD) is the most common form of degenerative dementia, yet currently available therapies such as cholinesterase inhibitors only offer modest symptomatic benefits. Given that AD is histopathologically characterized by the formation of extracellular plaques containing amyloid-β and intracellular neurofibrillary tangles consisting of hyperphosphorylated tau, many clinical trials over the course of the last few decades have aimed at clearing these two pathologies. Indeed, recent trials of monoclonal anti-amyloid-β antibodies have demonstrated good efficacy in reducing amyloid-β levels in the brain, resulting in FDA approvals; however, clinical benefits have been very modest with participants on active therapy continuing to decline at only a marginally reduced rate compared to placebo.^1,2^ This highlights the ongoing need to explore novel therapeutic approaches to treating AD.

An emerging strategy is focused ultrasound.^3^ At high intensity (high-intensity focused ultrasound), the modality generates heat and is used as an incisionless, FDA-approved surgical tool for treating essential tremor and tremor-dominant Parkinson’s disease.^4^ At low intensity, focused ultrasound used in a scanning mode (scanning ultrasound, SUS) exerts its effects without generating heat.^5^ SUS achieves neuromodulatory effects by delivering an acoustic pressure wave. SUS can also be deployed in combination with intravenously injected microbubbles (MBs) to (additionally) achieve transient blood–brain barrier opening (SUS^+MB^) and thereby, focused brain uptake of co-injected therapeutic agents, which are otherwise excluded from the brain (SUS^+MB+drug^).^3,5^ Upon applying SUS^+MB^ to amyloid-depositing APP23 mice, we achieved highly significant clearance of amyloid-β pathology and improved memory functions in three spatial memory tasks.^6^ Other groups similarly achieved amyloid-β clearance and functional improvements using this strategy.^7,8^

A question that arises is whether cognitive deficits can be restored without lowering amyloid-β levels.^9^ We previously addressed this question by treating senescent wild-type mice. Surprisingly, repeated SUS treatments, without the use of MBs and hence in the absence of blood–brain barrier opening (to distinguish the modalities, we termed this treatment SUS^only^, but we will be using SUS from here on for simplicity), restored long-term potentiation induction and improved spatial memory in a dose-dependent manner.^10^ As an underlying mechanism of SUS, we identified reductions in the density of perineuronal nets, increased neurogenesis and improved synaptic signalling, as well as NMDA receptor subunit changes indicative of long-term potentiation restoration. Extending these findings to APP23 mice, we again found that SUS improved spatial memory and other functions; interestingly, this occurred in the absence of any amyloid-β reductions.^11^ This finding mirrors our earlier work that removing endogenous tau protein in APP23 mice caused memory improvements in the absence of amyloid-β reductions.^9,12^ In the SUS study,^11^ we argued based on quantitative proteomics and functional MRI that this modality induced long-lasting functional changes that correlated with the observed improvement in memory.

These data prompted us to determine the safety and tolerability of SUS in humans. What we had to consider on this path is that mice and humans differ significantly in terms of their brain size and skull bone properties, the latter affecting attenuation. Ultrasound attenuation impacts the delivery of the required acoustic energy and thereby the intended bio-effects in brain tissue. We used sheep as a large animal model because their skulls are similar to those of humans in regard to thickness, porosity and curvature of the calvarium^13^ (and Song *et al*., Sci Rep., in press). Moreover, sheep possess the largest gyrencephalic brain amongst typical experimental animal species. Sheep enabled testing of human-scale prototypes to meet criteria of functionality, usability and ergonomics in humans; this ultimately led to the investigational UltraThera^Pilot^ device used here. In this process, the transducer was swapped for one with a lower centre frequency than what we had used in mice in order to reduce skull attenuation. This lower frequency, in turn, generated a lower acoustic radiation force which necessitated a compensatory increase in acoustic pressure. Because the resulting pressure was higher than that previously explored by other groups, we performed extensive preclinical safety testing in sheep (data unpublished) prior to initiating the human study. Here, the UltraThera^Pilot^ device was combined with a Brainsight image-guided neuronavigation system to enable targeting of specific brain regions and deployed into a first-in-human pilot study in participants with mild-to-moderate AD.

## Materials and methods

### Trial design

**Figure 1:**
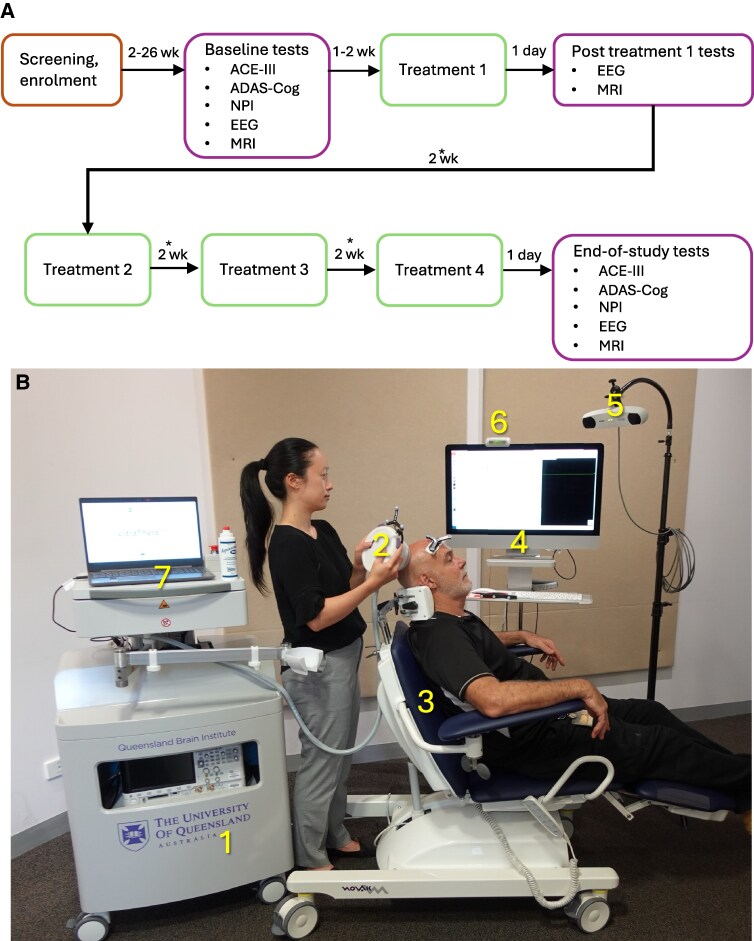
**Workflow of study visits and UltraThera^Pilot^ system components**. (**A**) Workflow of treatment and assessment visits. In addition to the in-person adverse event (AE) monitoring on treatment days, phone call reviews to monitor for AEs were conducted at Days +7, +21 and +35 (indicated by *). (**B**) UltraThera^Pilot^ system components. 1: UltraThera^Pilot^ trolley, 2: UltraThera^Pilot^ Probe, 3: Treatment chair with custom headrest, 4: Brainsight image-guided navigation system, 5: Brainsight guidance camera, 6: Remote indicator and 7: General purpose laptop. wk, week; ACE-III, Addenbrooke’s Cognitive Examination version III; ADAS-Cog, Alzheimer’s Disease Assessment Scale—Cognitive subscale; NPI, Neuropsychiatric Inventory.

**Figure 2:**
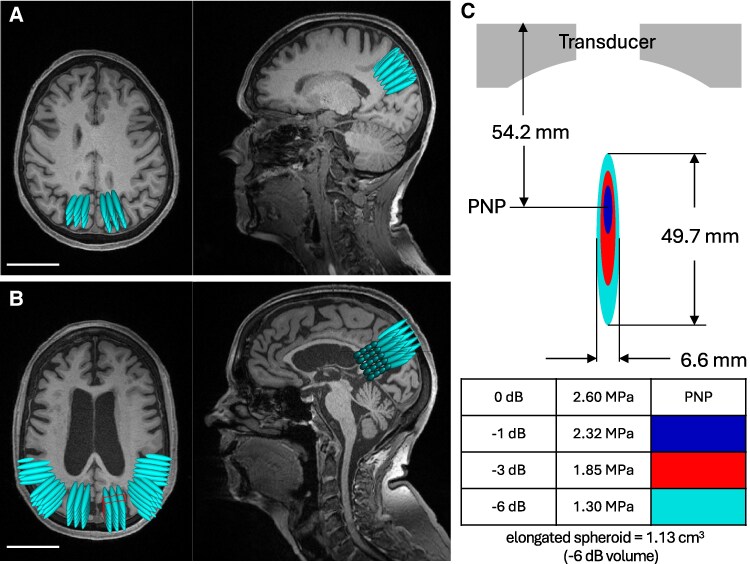
**Treatment planning**. Volumetric MRI scans from baseline visits were used to plan each participant’s treatment in the Brainsight navigation system. Blue spheroids denote individual targets and represent the −6 dB volume of the ultrasound transducer’s focal region. Axial (left) and sagittal (right) views shown as examples of (**A**) target volumes for Cohort 1 receiving 30 sonications per treatment visit (30 cm^3^, targeting the bilateral precuneus region) (**B**) and Cohort 2 receiving 100 sonications per treatment visit (100 cm^3^, bilateral temporo-parietal association cortex). (**C**) Focus profile measured in free field. MPa, megapascal; PNP, peak negative pressure. Scale bar (A,B): 50 mm.

The single-centre open-label pilot study was registered with the Australian New Zealand Clinical Trials Registry (ANZCTR) under registration number ACTRN1262200827730. Twelve participants with mild-to-moderate AD [mini-mental state examination (MMSE) score ≥10/30] were enrolled to test the safety and tolerability of repeated SUS treatments (Fig. 1A). Baseline EEG, MRI, and clinical measures of cognition and behaviour were followed by the first SUS treatment at Day 0, using the UltraThera^Pilot^ system (Fig. 1B) explained further below. On the next day, EEG and MRI were repeated. Three subsequent SUS treatments were delivered at fortnightly intervals, with the clinical measures, MRI and EEG being repeated 1 day after the final treatment. The 12 participants were divided into two cohorts: Cohort 1 (*n* = 4) who were planned to receive 30 sonications per treatment visit (30 cm^3^, targeting the bilateral precuneus/posterior cingulate region), followed by Cohort 2 (*n* = 8) who would receive 100 sonications per treatment visit (100 cm^3^, bilateral precuneus/posterior cingulate and lateral temporo-parietal association cortex) (Fig. 2A and B).

### Study participants

The participants were enrolled at the Memory and Cognitive Disorders Clinic at the Mater Misericordiae Hospital (Brisbane, Queensland, Australia). Each had a clinical dementia syndrome consistent with AD and confirmation of Alzheimer’s pathology using either cerebrospinal fluid biomarkers or amyloid-ligand positron emission tomography. Briefly, to be eligible, participants needed to be aged 50–85 years, have a regular caregiver/informant and be able to undergo MRI scanning. The key exclusion criteria included a history of bleeding disorders (including the presence of micro-haemorrhages on susceptibility-weighted MRI as assessed by an experienced neuroradiologist) and diseases of the skull or scalp that may have interfered with SUS delivery. The full inclusion and exclusion criteria are provided in Supplementary Table 1. Thirteen people were screened with one screen failure due to the diagnosis of a non-AD dementia. There were no dropouts during the study. The demographics of the 12 participants are summarized in Table 1.

**Table 1:** Participant demographics

	Cohort 1 *N* = 4	Cohort 2 *N* = 8	Total *N* = 12
Age mean (SD)	69.3 (6.3)	70.9 (7.7)	70.3 (7.0)
Sex (%)	3 F (75%)	3 F (38%)	6 F (50%)
MMSE mean (SD)	21.0 (6.9)	18.8 (4.3)	19.5 (5.1)
Height, cm mean (SD)	162.8 (3.3)	169.6 (13.8)	167.3 (11.6)
Weight, kg mean (SD)	62.6 (6.3)	74.9 (17.1)	70.8 (15.3)
BMI, kg/m^2^ mean (SD)	23.6 (1.7)	25.8 (3.4)	25.1 (3.1)

### Device description

The UltraThera^Pilot^ system is a novel device built under ISO 13485 guidelines. It consists of a free-standing, mobile trolley containing the SUS hardware and support systems, as well as a sonication check rig, an image-guided neuronavigation system and the participant chair (Fig. 1B). At its core, UltraThera^Pilot^ is a single-channel focused ultrasound transmitter with a signal chain consisting of a function generator, RF amplifier, impedance-matching transformer and spherically focused ultrasound transducer. Complementing this ultrasound system is an inbuilt fluid handling system that fills a silicone coupling cone with degassed, reversed osmosis water to provide acoustic coupling. During treatments, ultrasound gel was applied to the outside of the cone to complete coupling to the scalp.

Sonication targets were placed in grids with a pitch of 6 × 6 mm, and treatment volumes were marked by a 6.6 × 49.7 mm spheroid denoting the −6 dB contour of the transducer’s focal point (Fig. 2C). During treatments, the location of each sonication delivery was recorded in the Brainsight system, sampled in synchronization with the sonication. Participants were seated in an adjustable chair (Novak ENT, Novak M d.o.o., Komenda, Slovenia) equipped with a custom-made headrest.

The UltraThera^Pilot^ handheld probe included the ultrasound transducer (H117, Sonic Concept Inc., USA) driven at 286 kHz by an arbitrary waveform generator (AFG31022, Tektronix, Inc., USA) via a 40 W 50 dB gain radio frequency amplifier (240L, Electronics and Innovation, LTD, USA) and an impedance-matching network (Sonic Concept Inc., USA), an inflatable coupling cone connected to a fluidics system and control electronics necessary to allow positioning of the probe to the participant.

### Translating ultrasound parameters from mouse and sheep studies

We determined the ultrasound dose in the human study based on experimental work in sheep given their similarity in skull properties and brain anatomy,^13^ aiming to achieve a dose that in mouse studies had been shown to improve cognition^10,11^ (Table 2). Therefore, an external peak negative pressure (PNP) of 2.6 MPa was applied to five sheep, using a transducer operating at 286 kHz. The external PNP of 2.6 MPa is higher than the pressure applied in most previously reported human non-invasive brain stimulation (NIBS) trials, that ranged from 0.1 to 0.8 MPa, with one early BrainSonix study using a PNP of up to 1.985 MPa in free field.^14-17^ It is important to note that the neuromodulatory response has been shown to increase gradually as the PNP is being increased.^18-20^ The sheep study demonstrated safety as evidenced by the absence of damage both at a gross anatomy and histological level, including the absence of erythrocyte extravasation (petechiae) into brain tissue. Furthermore, no abnormalities were detected *in vivo* using MRI (T_2_-weighted, T_1_VIBE + gadolinium-contrast, susceptibility-weighted imaging and FLAIR sequences) (data not shown). We also performed Onda tank measurements of dissected sheep skulls (13 spots from 3 sheep), obtaining an attenuation of 64% (±5.8%), thereby arriving at an internal pressure of 0.9 ± 0.05 MPa, with the maximal derated PNP being 1.04 MPa. The other ultrasound parameters were 10 ms pulse length, 10 Hz pulse repetition frequency and 6 s sonication duration (Table 2), which is consistent with our preclinical work in mice. The above ultrasound parameters satisfied the ITRUSST and FDA guidelines.^21^

**Table 2:** Preclinical and clinical acoustic parameters in mice, sheep and humans

Species and clinical trial dose details (if applicable)	Frequency *f* (MHz)	Free field PNP (MPa)	Derated PNP (MPa)	Pulsing scheme (fixed acoustic parameters)			Acoustic radiation force index *fp^2^*	I_SPPA_ (W/cm^2^)
				Pulse repetition frequency (Hz)	Pulse length (ms)	Sonication duration (s)		
Mouse	1.0	0.7	0.57^a^	10	10	6	0.325	11.0
Sheep	0.286	2.6	≤1.04^b^	10	10	6	≤0.309	≤37.5
Human—100% dose	0.286	2.6	≤1.04^b^	10	10	6	≤0.309	≤37.5
Human—75% dose	0.286	1.95	≤0.78^b^	10	10	6	≤0.174	≤21.1
Human—50% dose	0.286	1.3	≤0.52^b^	10	10	6	≤0.077	≤9.4

^a^Calculated based on an average 18% skull attenuation in mice;.

^b^Calculated based on an estimated 64% average skull attenuation of bone thicknesses between 3.80 and 7.70 mm.

### SUS dosage

A PNP of 2.6 MPa is relatively high for NIBS, raising a potential tolerability issue. Thus, a descending dose protocol was formulated with the addition of 75% (PNP = 1.95 MPa), and 50% (PNP = 1.3 MPa) doses in case the 2.6 MPa dose were poorly tolerated. As the PNP was the only parameter varied, this was termed the ‘dose’ for the trial. Each participant was to commence at 2.6 MPa. The protocol allowed for the dose to be dialled down in real time during a treatment session to 1.95 MPa and again to 1.3 MPa, depending on how well each dose was tolerated. Failure to tolerate the 1.3 MPa dose was deemed to indicate that a participant could not tolerate the procedure and would constitute termination of their participation in the study.

### MRI-informed SUS treatment procedure

To accurately guide the ultrasound probe to the pre-planned targets in the brain, an image-guided navigation system was used (Brainsight TMS, Rogue Research, Montreal, Canada). The Brainsight system was used unaltered except for a custom calibration block adaptor. Baseline volumetric (0.8 mm isotropic resolution) T_1_-weighted MRI scans were used to plan the sonication locations in the Brainsight software. The 30 sonications (∼30 cm^3^ of brain tissue) for Cohort 1 targeted the bilateral precuneus region. The 100 sonications (∼100 cm^3^) for Cohort 2 targeted the bilateral precuneus and bilateral temporo-parietal association cortex (Fig. 2).

At each treatment visit, the participants had their head shaved to ensure air bubble–free coupling of the ultrasound probe to the scalp (wigs were available on request). The participants were then seated in the reclinable chair with their head supported by a neck rest.

Immediately before treatment, fiducial markers were applied to the participant’s forehead, and they were registered as per standard Brainsight workflow. Ultrasound gel was then applied to the probe. The probe was placed on the participant’s scalp and positioned to the level at which the focus of the ultrasound was at the required depth for the target brain region. The interface between the probe and the scalp was directly visualized throughout the procedure to ensure that no air bubbles were introduced that would interfere with the coupling. The probe was then aligned to the target position as indicated on the Brainsight display. SUS was applied (with each sonication lasting 6 s), and the probe was then slid across the scalp to the next position of the target grid.

### Safety monitoring

At the beginning of each visit, the participant and caregiver were asked to report any new symptoms, diagnoses or alterations to concomitant medications. Vital signs were recorded immediately before and after each sonication session. During the sonication session, the treatment team was in continuous discussion with the participant to ensure their comfort. Seven days after Treatment visits 1, 2 and 3, a phone call follow-up was conducted to monitor for possible adverse events (AEs). The neuroradiologist examined the MRI scans acquired 24 h post Treatment 1 and at the end-of-study in comparison to the baseline scan for the emergence of any treatment-related abnormalities (such as oedema or haemorrhage).

### Primary and secondary end-points

Primary end-points were safety and tolerability of the SUS procedure and feasibility of the clinical workflow.

Secondary end-points comprised measures of cognition using the Addenbrooke’s Cognitive Examination version III (ACE-III)^22^ and the Alzheimer’s Disease Assessment Scale—Cognitive subscale (ADAS-Cog),^23^ and informant reported behavioural and psychological symptoms of dementia (BPSD) using the 12-item Neuropsychiatric Inventory (NPI) test.^24^

Further secondary end-points—resting-state EEG as well as resting-state and diffusion-weighted MRI—were aimed at identifying evidence of perturbations in these measures that might offer biomarker evidence of target engagement. These analyses specifically explored evidence for change from baseline to the follow-up sessions after the first and fourth SUS treatments.

### MRI and EEG acquisition and processing

MRI had three purposes in the study: (i) ensuring eligibility and safety monitoring, (ii) positioning of the sonication targets and (iii) exploratory analyses of diffusion and resting-state functional data to assess for signal alterations that might represent target engagement. EEG was similarly explored as a potential physiological marker of target engagement. The full details of acquisition and processing for MRI and EEG data are found in Supplementary material and Methods (Supplementary Table 2). The awake resting-state EEG was always performed at the same time of day and immediately prior to the MRI scan.

### Statistical analysis

We summarized the participants’ demographics using descriptive statistics, expressed as mean ± standard deviation. Paired *t*-tests were performed on EEG measures, ACE-III and ADAS-Cog, and Wilcoxon rank-sum test was used for NPI and NPI caregiver distress scores. All tests were performed in SPSS or Matlab, with a significance level set at 0.05.

### Ethics

The study was approved by the Bellberry Human Research Ethics Committee (Ref: 2021-11-1320) and registered with the ANZCTR (12622000827730). All participants and their caregivers gave voluntary, written informed consent. The trial was conducted in accordance with the Declaration of Helsinki. The study team had been trained in, and adhered to, the principles of Good Clinical Practice.

## Results

### Primary end-point—feasibility

Of the 12 participants who met eligibility criteria, all completed all aspects of the study in accordance with the protocol.

With the operator having gone through several training sessions, the SUS clinical workflow demonstrated feasibility throughout the trial. The UltraThera^Pilot^ device reliably delivered 3680 sonications over 48 treatment sessions in total. Minor refinements were made as the trial progressed to improve ergonomics for the ultrasound operator, which increased the treatment speed and improved the system setup efficiency.

The UltraThera^Pilot^ treatment was brief with sonication sessions taking ∼7 min to complete for Cohort 1 and 24 min for Cohort 2. Participants were typically on-site for under 45 min. This was achieved because our coupling system allowed for the probe to be held stably in place for the duration of each 6 s sonication while also allowing to slide smoothly from each target position to the next on the shaved scalp. Targeting accuracy was high according to the Brainsight sampling, with errors generally under 0.5 mm.

### Primary end-point—safety

Collectively, there was neither alteration to the mental state nor any significant change to vital signs in association with the acute delivery of the SUS treatment in any participant. The neuroradiologist’s visual read of the MRI scans performed after the first, and final, treatment sessions identified no changes from baseline scan in any participant (data not shown).

There was only one treatment emergent adverse event that was deemed related to the ultrasound procedure: one participant developed scalp irritation precipitated by head shaving and diaphoresis from the wearing of a wig. There was one serious AE, deemed unrelated to the SUS treatment: one participant with a known history of abdominal problems attributed to diverticular disease presented with an acute small bowel obstruction necessitating admission to hospital. The episode was resolved with conservative measures. The complete list of treatment emergent adverse events is shown in Supplementary Table 3.

### Primary end-point—tolerability

The initial 100% dose of 2.6 MPa in free field was associated with pain of sufficient severity to cause flinching in all four participants of Cohort 1 (three at the first treatment visit and one at the second visit). Accordingly, after several sonications at the 100% dose, the dose was titrated down to 75% (1.95 MPa). At this dose, each participant tolerated the remainder of the procedure without difficulty, and the 1.95 MPa dose was maintained at subsequent treatment visits. Because this occurred in all participants of Cohort 1, a protocol amendment was submitted to the Ethics Committee to abandon the 2.6 MPa dose. Thereafter, all remaining participants (Cohort 2) were treated at the 1.95 MPa PNP dose, with none needing to down-titrate further. At 1.95 MPa, some participants experienced some minor discomfort with some sonication spots, but this was never severe; no participants ever requested to discontinue the session. Participants also sometimes reported that they could hear sound when the sonication was delivered.

### Exploratory secondary end-points—behavioural measures

**Figure 3:**
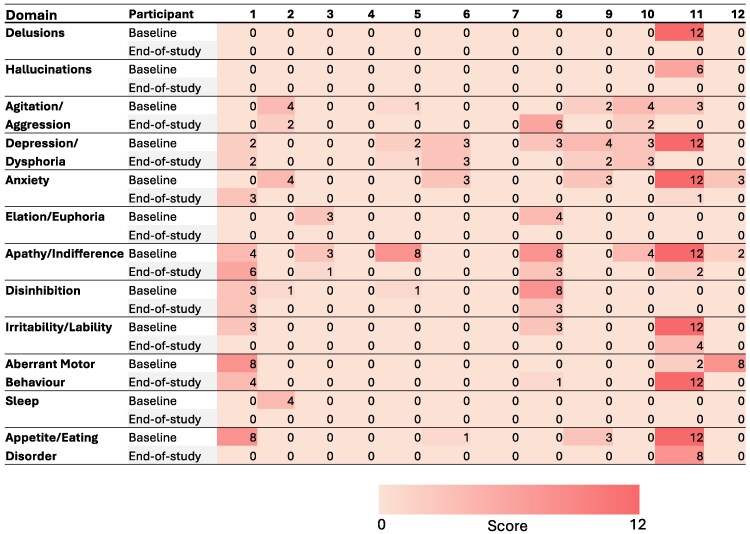
**NPI raw score heatmap**. Ten of the 12 participants show reduced total scores from baseline to end-of-study. Similarly, caregiver distress, as measured by the NPI caregiver distress scale, decreased from baseline to the end of the study in 8 of the 12 participants, with 1 increasing and 3 not changing.

There were no significant changes in cognitive assessments as measured by the ACE-III and ADAS-Cog from baseline to end-of-study (Table 3). In contrast, there was a significant improvement in BPSD as measured by the 12-item NPI. Similarly, caregiver distress, as measured by the NPI caregiver distress scale, decreased from baseline to end-of-study, a change that approached statistical significance (Fig. 3).

**Table 3:** Baseline and end-of-study cognition and behaviour scores^a^

Test	Baseline	End-of-study	
ACE-III mean (SD)	57.1 (19.7)	55.8 (21.5)	*T* = 0.8*, P* = 0.4^b^
ADAS-cog mean (SD)	36.7 (13.7)	38.3 (13.4)	*T* = −0.64, *P* = 0.5^b^
12-item NPI median (IQR)	12 (16.5)	2 (10.8)	*Z* = −2.76, *P* = 0.006^c^
NPI caregiver distress median (IQR)	7 (8.5)	2.5 (5.3)	*Z* = −1.96, *P* = 0.05^c^

^a^Lower scores denote worse performance on ACE-III and higher scores denote worse performance on all other measures. SD, standard deviation; IQR, inter-quartile range.

^b^Paired *t*-test.

^c^Wilcoxon signed rank test.

### Exploratory secondary end-point—EEG

A comparison of EEG recordings at the start and end of the trial revealed no significant changes in the typical EEG indicators associated with AD, such as reduced alpha spectral power, slower alpha frequency and reduced alpha coherence. There were no significant differences in alpha peak frequency between sessions either across the region of interest (ROI) (all *P*-values > 0.85) or when analysing each electrode separately and using cluster permutation to correct for multiple comparisons (all *P*-values > 0.22) (Supplementary Fig. 1A–F). Alpha power also showed no significant differences between sessions either across the ROI (all *P*-values > 0.31) or when analysing each electrode separately and correcting for multiple comparisons with cluster permutation. The aperiodic-corrected oscillatory Delta, Theta, Alpha and Beta spectra (Supplementary Fig. 2A–D) were used to calculate the spectral power ratio (Supplementary Fig. 2E). No significant differences between sessions were observed at the ROI or when analysing each electrode separately and correcting for multiple comparisons with cluster permutation. These findings suggest that standard measures of brain electrical activity remained stable throughout the study period.

**Figure 4:**
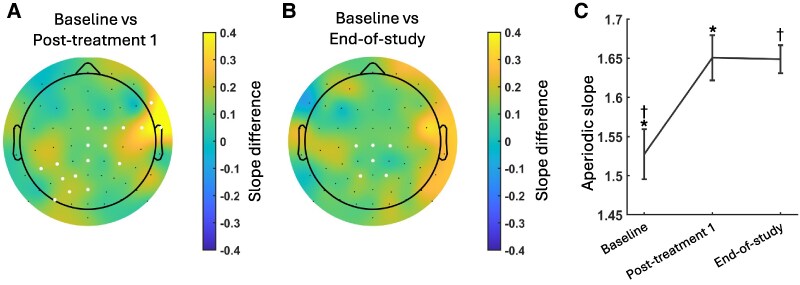
**EEG—significant differences in aperiodic slope**. (**A**, **B**) Topographies showing the difference in aperiodic slope of baseline versus post Treatment 1 and baseline versus end-of-study. White dots indicate electrodes with significant differences as determined by repeated-measures *t*-tests, corrected for multiple comparisons with cluster-based permutation. (**C**) Aperiodic slope averaged for each session. Error bars represent within-participants standard error of the mean. Aperiodic slopes were compared between the three times using paired-samples *t*-tests (*N* = 12 participants). The baseline session (pretreatment) had significantly flatter slopes than the two later sessions (pretreatment versus Post-treatment 1: *t*_11_ = 2.31, †*P* = 0.041; pretreatment versus end-of-study: *t*_11_ = 2.99, *P = 0.012). There was no difference in between Post-treatment 1 and the end-of-study (*t*_11_ = 0.36, *P* = 0.725).

By contrast, there were some noteworthy changes in aperiodic EEG content (Fig. 4A and B). Aperiodic slopes (exponents) were significantly steeper across the parietal ROI between the baseline session (pretreatment) and the two later sessions (pretreatment versus follow-up: *t*_11_ = 2.31, *P* = 0.041; pretreatment versus end-of-study: *t*_11_ = 2.99, *P* = 0.012). There was no difference in slopes between follow-up and end-of-study EEG sessions (*P* = 0.725). This pattern was borne out in a cluster permutation test, which showed a single significant cluster in both the baseline versus follow-up comparison (*P* < 0.001) and the baseline versus end-of-study comparison (*P* < 0.001). There were no significant clusters in the follow-up versus end-of-study comparison.

### Exploratory secondary end-point—MRI

Cortical volume and diffusion-weighted metrics in both treatment and control regions showed no significant changes between baseline and end-of-study (*P-*values > 0.11, uncorrected for multiple comparisons) (Supplementary Fig. 3A–C). Additionally, both control and treatment regions exhibited no change in these two metrics over time. Tract-based spatial statistics analysis of the diffusion imaging revealed no significant white-matter changes. No significant changes in functional activity were observed comparing end-of-study with baseline within treatment ROI (*P-*values = 0.17), control ROI (*P-*values = 0.63) or frontal default mode network control ROI (*P-*values = 0.83). Similarly, when comparing functional activity between treatment and frontal control ROI (*P*-value = 0.44) or treatment and default mode network control ROI (*P*-value = 0.4) or the two control ROIs (*P*-value = 0.8), no significant differences were found (Supplementary Fig. 3A). Connectivity changes due to treatment were also not significant when comparing treatment ROIs to control ROIs (*P-*values = 0.9) (Supplementary Fig. 3B) or to frontal default mode network control ROIs (*P-*values = 0.65) (Supplementary Fig. 3C).

## Discussion

### Feasibility, safety and tolerability of SUS

Ultrasound is increasingly being explored for NIBS. Our study used an external PNP of 1.95 MPa and I_SPPA_ = 21.1 W/cm^2^, a dose that was safely tolerated by 12 study participants having AD with a mean MMSE of 19.5 (±5.1). To the best of our knowledge, the derated PNP used in this study was higher than that used in any previous focused ultrasound neuromodulation studies.^14-17,25-27^ The open-label study—comprising two cohorts, targeting 30 cm^3^ in Cohort 1 (*n* = 4) and 100 cm^3^ in Cohort 2 (*n* = 8), with these treatments repeated four times at fortnightly intervals—was deemed safe, feasible and tolerable, the latter reflected by the fact that there were no dropouts in the study and all participants completed all scheduled sonications.

Typical treatments required only one coupling per side of the shaved head (50 sonications in Cohort 2), thereby increasing speed, efficiency and reliability of the SUS delivery. Although well-tolerated, we foresee that in the future, shaving can be omitted in several treatment scenarios. When not traversing wide areas of the head, or in the absence of time constraints, sufficient coupling can be achieved by flattening and wetting hair, applying gel and manually removing any air bubbles. Interestingly, there were no serious AEs attributable to the procedure and no evidence of imaging abnormalities being produced. The only treatment emergent adverse event attributable to the procedure was related to the requirement for scalp shaving rather than the delivery of ultrasound.

The starting dose with a PNP of 2.6 MPa (100%) was not tolerated by the first four participants because of discomfort at the site of sonication and was therefore abandoned. There were no tolerability issues at 1.95 MPa PNP (75%) and, therefore, the lowest planned dose (PNP = 1.3 MPa, 50%) was never required to be administered. Of note, a PNP of 1.95 MPa is still higher than what has been used in the majority of other neuromodulation studies.^14,16^ Moreover, several studies including ours support the notion of a gradual dose response to the acoustic radiation force (ultrasound intensity). This concept has been discussed^20^ and is experimentally supported by studies in mice.^11,18^

Participants typically could hear and/or feel some sonications, an important detail to address when designing future sham-controlled clinical trials. The UltraThera^Pilot^ device proved to be reliable and user friendly throughout the trial, with no technical failures. Image-guided navigation allowed for targeting accuracy in delivering the sonications and for the procedure to be done reasonably fast and in a simple manner. Only minor device upgrades (i.e. improved sonication button placement for better ergonomics) were made during the trial. Over the course of the study, a total of 3680 sonications, each 6 s long, were delivered, using a handheld device that was moved in a scanning, i.e. SUS mode, and positioned by the sonographer using the Brainsight image-guided navigation system. Regarding targeting accuracy, although a 3 mm positioning error was allowable under our trial protocol, errors for individual sonications were typically recorded by Brainsight as being 0.5 mm or below. Targeting errors were further mitigated by the fact that we treated multiple, juxtaposed spots in a sequence, and not just a single spot. Also, at treatment planning, the targets were placed on each participant’s actual volumetric MRI scan to ensure sonications were always being delivered to brain tissue and not into *ex vacuo* CSF spaces. Furthermore, positions were recorded at the start of each 6 s sonication, and any potential movement afterwards was minimized by stable coupling of the probe to the participant’s head.

### Effects of SUS on cognitive and behavioural readouts

As exploratory secondary end-points, cognitive and behavioural measures were included in our SUS study. While we did not find changes in cognitive test scores, there were significant improvements in BPSD and caregiver distress ratings as measured by the NPI. While very encouraging, it is important to stress that this was an open-label study and confirmation that SUS can improve dementia-related BPSD will need sham-controlled trials. Interestingly, a recent meta-analysis of transcranial magnetic stimulation clinical trials in dementia suggested that, depending on the protocol employed, transcranial magnetic stimulation can lead to improvements in BPSD.^28^ By extension, there may also be an ideal combination of ultrasound parameters for achieving improved behavioural outcomes. For example, several studies have demonstrated that in particular one parameter, the pulse repetition frequency, can affect functional outcomes, with higher values more likely causing excitatory and lower values inhibitory effects.^19,29^ In the human study presented here, however, we used ultrasound parameters that in mice were associated with improved cognitive and behavioural outcomes (Table 2).

Notably, the NPI scores revealed considerable interindividual variability in the treatment response. This heterogeneity could reflect a range of underlying factors, including differences in baseline severity and profile of neuropsychiatric symptoms, differences in opinions across the informants, the extent to which targeted brain regions were engaged during SUS or individual variability in susceptibility to ultrasound-induced neuromodulation. Other contributors may include differences in concomitant medications, comorbidities or stage of disease progression, all of which can shape behavioural outcomes in dementia. These observations underscore the necessity for a future sham-controlled trial.

### Effects of SUS on resting-state EEG and MRI biomarkers

EEG as well as resting-state functional and diffusion-weighted MRI were included as exploratory secondary end-points aimed at identifying evidence of perturbations that might offer biomarker evidence of target engagement. These analyses specifically explored evidence for a change from baseline to the scanning sessions after the first and fourth SUS treatments. MRI examined for subtle changes at the treatment sites in cortical thickness due to swelling, changes in water diffusivity with diffusion-weighted imaging, and changes in resting-state connectivity. These analyses were all negative. Interestingly, however, EEG revealed a steeper aperiodic slope in post sonication sessions when compared with the baseline EEG. This change in aperiodic slope, localized to electrodes placed on the region of the head that was treated with SUS, indicates some level of target engagement. This implies that the treatment may have had localized effects on brain dynamics, despite the absence of changes in more traditional EEG measures. Steeper aperiodic slopes have been reported to correspond to a shift in the neuronal balance of excitation to inhibition towards increased inhibition^30,31^ and may indicate an increase in inhibition localized to the region of the brain that received treatment.^32^ Moreover, flatter aperiodic slopes have been associated with older age and poor cognitive efficiency,^33,34^ providing further evidence that SUS treatment may potentially benefit cognition. It is important to note that aperiodic changes in EEG have been documented following other neuromodulation techniques, such as transcranial magnetic stimulation and electroconvulsive therapy.^35,36^ These types of alterations in brain activity highlight the complex nature of the brain’s response to NIBS and suggest that different aspects of EEG signals can reveal different treatment effects. Overall, while the study found no significant changes in the typical EEG indicators of AD, the observed alterations in aperiodic EEG content suggest potential engagement with the targeted brain region. This finding opens avenues for further exploration into the effects of neuromodulation on brain activity and underscores the need for ongoing research in this area.

### Limitations

There are several limitations to our study. The first is in relation to the 286 kHz centre frequency used in the human trial. Most of our foundational preclinical work in mice used a transducer operating at 1 MHz. A side-by-side comparison of a 286 kHz and 1 MHz paradigm had revealed that after adjusting the external pressure to arrive at an equivalent estimated derated PNP, 1 MHz was more effective than 286 kHz in improving cognitive functions in amyloid-depositing APP23 mice.^11^ However, because at 1 MHz the human skull causes considerable attenuation and the sonicated volume becomes smaller, meaning that less brain volume is stimulated per sonication, we opted for a 286 kHz frequency. To address this potential confound, rather than using a derated pressure in humans that was equivalent to that used in mice, in our clinical study, we used a broadly equivalent acoustic radiation force for the 100% (2.6 MPa) dose. A second variation was the use of a 75% dose throughout most of the trial. While this had been taken into consideration in the trial design and only affected the smaller Cohort 1, representing approximately 1% of the total sonication events, this could present a potential confound in the interpretation of the exploratory analyses for biological effects. While this cannot be formally ruled out, our preclinical work suggests a cumulative treatment effect. In our SUS study in senescent mice, we have shown that a single treatment had no effect on spatial memory in the active place avoidance test, but that improvements were seen as the number of treatments were increased.^10^ By extension, we would argue that in the clinical study presented here, the early adjustment of the dose from 100 to 75% had negligible effects. Regarding the frequency, a follow-up trial could explore a frequency sitting between 286 kHz and 1 MHz. A potentially larger confound to the interpretation of the exploratory analyses of biological effects was that Cohort 1 received 30, whereas Cohort 2 received 100, sonications per treatment session. In hindsight, limiting Cohort 1 to only 30 sonications per treatment session was excessively cautious in that no participant in Cohort 2 had even the slightest difficulty in tolerating 100 sonications per session. It is important to note, however, that while combining these two cohorts may have had an influence on interpretation of the exploratory secondary end-points, the primary end-point was safety and tolerability for this first-in-human study. As such, the cautious approach of escalating the number of sonications from Cohort 1 to 2 for safety reasons was deemed more important that having a perfectly uniform dataset for the exploratory analyses. Secondly, although the significant improvements in BPSD and associated caregiver distress ratings are very encouraging, the data need to be interpreted with caution as possible placebo effects until sham-controlled studies are available. Thirdly, although the handheld treatment procedure developed here was quick and accurate, with up to three participants treated in a single day, further ergonomic refinements may be considered if one were to treat more subjects and/or brain areas >100 cm^3^ with this system. Finally, the variability in the attenuation of SUS as expected across the different sonicated spots was not considered in our treatment planning, which would have resulted in an inconsistent neuromodulatory effect between spots and participants. Numerical modelling such as k-Wave simulation,^37^ MR thermometry or MR acoustic radiation force imaging^38,39^ would be useful to deliver a uniform ultrasound dose.

## Conclusion

In this first-in-human safety, feasibility and tolerability study, we demonstrated that AD participants safely tolerate sonications of up to 100 cm^3^ of brain volume at a PNP of 1.95 MPa using a 286 kHz transducer and that the procedure can be performed within a reasonably short timeframe. The protocol was highly feasible and suitable even for more advanced AD participants displaying BPSD. A next step may be treating areas implicated in symptoms such as BPSD with SUS, by designing a sham-controlled efficacy study and applying the protocol to a larger trial cohort. Given the frequency considerations discussed above, the choice of ultrasound parameters may require further investigation, factoring in our preclinical studies^11^ and the clinical data presented here. More generally, we posit that in the future, different low-intensity ultrasound modalities may be deployed for the treatment of AD other than SUS that has a potential use as an emerging NIBS modality for symptomatic therapy. We foresee the utility of using SUS^+MB^  ^+^  ^drug^, i.e. low-intensity ultrasound that delivers drugs focally through a transiently opened blood–brain barrier, a strategy informed by an increasing number of preclinical and clinical studies.^3^

## Supplementary Material

fcaf445_Supplementary_Data

## Data Availability

The data that supports the findings of this study are available upon written request. Scripts used for EEG and MRI analysis can be found at: https://github.com/AnthMHarris/SUSinAD.
