# Supplementary material for: A pilot safety and tolerability study of scanning ultrasound as a neuromodulation therapy in Alzheimer’s disease
Source: Brain Commun. 2025 Dec 15;7(6):fcaf445. doi: 10.1093/braincomms/fcaf445 (PMC12703879; doi:10.1093/braincomms/fcaf445)
Supplement: fcaf445_Supplementary_Data [file fcaf445_supplementary_data.pdf]

Supplementary Material

A pilot safety and tolerability study of scanning ultrasound as a neuromodulation therapy in Alzheimer's disease

Peter J Nestor,[1,2,4,*] Matthew Pelekanos,[1] Gerhard Leinenga,[1] Jae Song,[1] Wendy Lee,[1] Gina Richter-Stretton,[1] Caitlin McElligott,[2] Amir Fazlollahi,[1] Jason B Mattingley,[3,4,5] Anthony Harris,[3] Henry Beale,[3] Jennie Roberts,[6] Rachel de las Heras,[1,7] and Jürgen Götz[1,4,*]

[1] Clem Jones Centre for Ageing Dementia Research, Queensland Brain Institute, The University of Queensland, Brisbane 4072, Australia
[2] Mater Public Hospital, South Brisbane 4101, Australia
[3] Queensland Brain Institute, The University of Queensland, Brisbane 4072, Australia
[4] NHMRC Centre of Research Excellence in Mechanisms in NeuroDegeneration – Alzheimer's Disease, The University of Queensland, Brisbane 4072, Australia
[5] School of Psychology, The University of Queensland, Brisbane 4072, Australia
[6] Radiology department, Royal Brisbane and Women's Hospital, Brisbane 4006, Australia
[7] Current address: Ceretas Pty Ltd, Brisbane 4000, Australia

Correspondence:

* Peter Nestor (p.nestor@uq.edu.au, ORCID 000-0002-5860-5921); Jürgen Götz (j.goetz@uq.edu.au, ORCID 0000-0001-8501-7896)

Supplementary Data

Supplementary Material and Methods

1. MRI analysis

MRI included 3D volumetric T1- and T2- weighted sequences; SWI; FLAIR; diffusion weighted and resting state functional MRI (rsfMRI) images. The MRI data served four purposes: (i) to determine eligibility according to the inclusion/exclusion criteria; (ii) to monitor safety; (iii) to plan the targets and guide the delivery of sonication treatment using the T1-weighted volumetric scan; and (iv) exploratory analyses looking for evidence of target engagement with rsfMRI and diffusion-weighted MRI. Safety monitoring and reporting for eligibility were performed by an experienced neuroradiologist.

2. MRI acquisition protocol

Each participant underwent a neuroimaging session on a 3.0T Siemens Prisma scanner (Siemens Healthineers, Erlangen, Germany) with a 64-channel head coil (software version

VE11). The following sequences were acquired, with parameters listed in **Supplementary Table 2**:

(i) A 3-Dimensional Magnetization-Prepared Rapid Gradient-Echo Imaging (MPRAGE) sequence was acquired to assess anatomical structure and brain atrophy, and to plan the SUS treatment.

(ii) T2-weighted isotropic turbo spin-echo (SPACE) images to assess perivascular space and lesions.

(iii) Multi-shell diffusion-weighted image (DWI) acquisition included 9 non-diffusion weighted images (b = 0 s/mm^2) as well as 27 (b = 1,000 s/mm^2) and 62 (b = 2,500 s/mm^2) unique directions. A B0 image was also acquired to correct for geometric distortion.

(iv) T2-weighted FLAIR images were included to assess white matter hyperintensity burden and evidence of signal change at the sites of sonication.

(v) Susceptibility-weighted images (SWI) were included to assess cerebral micro-haemorrhages.

(vi) Resting-state functional MRI (rsfMRI) data were acquired using a T2*-weighted gradient-echo echo-planar imaging (EPI) sequence, optimized for blood-oxygen-level-dependent (BOLD) contrast. The acquisition duration was 6:09 minutes, during which 490 volumes were acquired, with participants instructed to remain still.

3. MRI processing and analysis

The FreeSurfer 7.2.0 software was used to automatically segment and parcellate T1W scans, generate surface meshes, cortical thickness and regional brain volumes. The 15 mm-smoothed cortical thickness maps were transformed into z-scores to generate a heat map of cortical thinning for each participant in comparison to n = 31 healthy control participants. The regional volumes were scaled using total intracranial volume (ICV) for group comparison.

Diffusion images underwent denoising, motion correction across directions via rigid registration, susceptibility distortion correction, outlier detection/replacement, resampling, and intensity inhomogeneity correction. Diffusion tensor imaging (DTI) metrics—fractional anisotropy (FA), mean diffusivity (MD), axial diffusivity (AxD), and radial diffusivity (RD)—were estimated using DTIFIT in FMRIB's Software Library (FSL) [1].

rsfMRI preprocessing included motion correction (MCFLIRT), brain extraction (HD-BET), spatial smoothing (6 mm FWHM), and temporal filtering (high-pass, 100 s cutoff) using FSL 6.

Changes in cortical volume and DWI metrics before and after treatment were assessed using mixed-effects models in both treatment and control ROIs. Tract-Based Spatial Statistics (TBSS) was used to compare fractional anisotropy (FA) and mean diffusivity (MD) between baseline and end-of-study (EOS) time points.

Functional activity was evaluated using temporal standard deviation, while seed-based functional connectivity analysis was performed with the treatment region as the seed. Additionally, the medial prefrontal cortex (mPFC), a key component of the Default Mode Network (DMN), was included as a control region. A one-sample t-test was conducted to evaluate the percentage of change in activity attributed to treatment, while a paired t-test was used to compare the activity differences between treatment and control ROIs. Additionally, a paired t-test assessed differences in connectivity between pre- and post-treatment.

4. EEG acquisition protocol

EEG was used as an exploratory analysis to search for evidence of target engagement after the first and fourth SUS treatments compared to baseline. Data collected from the EEG included average power spectrum, aperiodic slope, alpha peak frequency, spectral power ratio (SPR), and average spectral coherence.

At the start of each EEG session, prior to EEG recording, participants were briefed about the procedure and what they should expect. They were fitted with an EEG cap, and the electrode locations were recorded relative to nasion and left and right helix-tragus junction references using a Polaris Spectra optical tracking system. Resting-state EEG recordings were taken in three five-minute blocks as participants sat with their eyes open, maintaining fixation on a central point displayed on a VIEWPixx /3D monitor (VPixx Technologies, Canada). Between each block, participants were given a self-paced break and were reminded of the importance of being still and maintaining fixation.

Continuous EEG data were recorded with a 64-electrode BioSemi ActiveTwo system, digitized at 1,024 Hz with a 24-bit A/D conversion. The 64 Ag/AgCl electrodes were arranged by the international standard 10-10 layout using a nylon head cap [2]. All scalp electrodes were referenced to the Common Mode Sense electrode during recording, with the Common Mode Sense and Driven Right Leg electrodes as the ground electrodes. Blinks were recorded with bipolar horizontal electro-oculographic (EOG) electrodes at the outer canthi of both eyes, and bipolar vertical EOG electrodes above and below the left eye, with bilateral mastoid electrodes as import references.

5. EEG processing and analysis

The EEG data were down-sampled to 256 Hz and band-pass filtered from 0.3 to 100 Hz, then separated into 100 x 3-second epochs for each 5-minute recording period, for a total of 300 epochs per session (baseline, follow up, and end-of-study). Bad channels, defined as channels with Kurtosis scores more than five standard deviations from the mean of all channels, were rejected from analysis. An average of 2.77 (SD = 2.01) channels were excluded per participant per session. Data were re-referenced to the average of all electrodes, and the average voltage for the entire epoch was subtracted from each channel as a baseline. Epochs containing absolute voltages greater than 1000µV were detected and removed as likely containing artifacts. The data were then analysed with infomax independent components analysis (ICA), and artifactual components such as blink- and eye-movement artifacts, muscle artifacts, and electrode noise, were removed based on visual inspection. Following ICA cleaning, the channels that had previously been rejected due to high kurtosis were interpolated with spherical spline interpolation. For all analyses, EEG indicators were calculated for each session separately at each electrode, and also averaged across a region of interest (ROI) centred on the central-parietal electrodes (Cz, C1, C2, CPz, CP1, CP2, Pz, P1, P2) broadly corresponding to the site of parietal SUS stimulation. For each individual analysis, data-points outside the bounds of ±3 inter-quartile ranges were excluded as outliers.

The average power spectrum for each participant in each session was calculated using multi-taper power spectral density estimates with a time-half bandwidth product of 4 applied to each 3-second epoch, which was then averaged across epochs. These spectra were analysed using the specparam package [3] for Python to quantify the aperiodic exponent (referred to here as 'slope') and offset as well as the properties of any neural oscillations (amplitude, peak frequency, bandwidth). Consistent with past findings [3], aperiodic offset and slope were strongly correlated ($r > .95$ within each session), indicating these measures were redundant. As such, aperiodic offset was excluded from further analysis.

We performed between-sessions comparisons using several EEG measures that have previously been associated with AD (for a review, see [4]). These included aperiodic slope,

alpha power and peak frequency, and the spectral power ratio (SPR). SPR reflects the ratio of power observed in low versus high frequencies of the EEG power spectrum: SPR = $(\alpha+\beta)/(\delta+\theta)$. For this analysis, the various frequency bands were defined as follows: Delta (δ) at 3–4 Hz, Theta (θ) at 4–8 Hz, Alpha (α) at 8–13 Hz, and Beta (β) at 13–30 Hz. SPR has been shown to be a sensitive measure that differentiates people with AD from healthy controls [5].

Another common EEG measure associated with AD is the average spectral coherence in the α-band across the scalp, which can indicate changes in the coordination of communication across brain networks [4]. To compare α-band spectral coherence between sessions, we bandpass filtered each epoch from each session between 8-13 Hz with a finite impulse response (FIR) filter, with a transition width of 25% of the boundary frequencies and a filter order of three times the number of samples per cycle of the lowest frequency (i.e., filter order = 3*(256/6) = 128). The complex-valued representation of the data was computed using a Hilbert transform, and spectral coherence was computed between 0.5-2.5 s within each epoch, to avoid the influence of edge artefacts. Spectral coherence was then averaged across epochs within each recording session.

Supplementary figures

Supplementary Figure 1

EEG - Alpha peak frequency. (A-C) Topographies of mean alpha peak frequencies observed in the EEG data for each session, and (D-F) differences between sessions. No significant modulations of alpha peak frequency were observed at any channel between any pair of sessions (assessed via cluster-permutation t-tests. All cluster mass < 2.89, all p > .215. N = 12 participants). Post-treatment 1 = one day after SUS (scanning ultrasound) treatment 1, End-of-study = one day after SUS treatment 4.

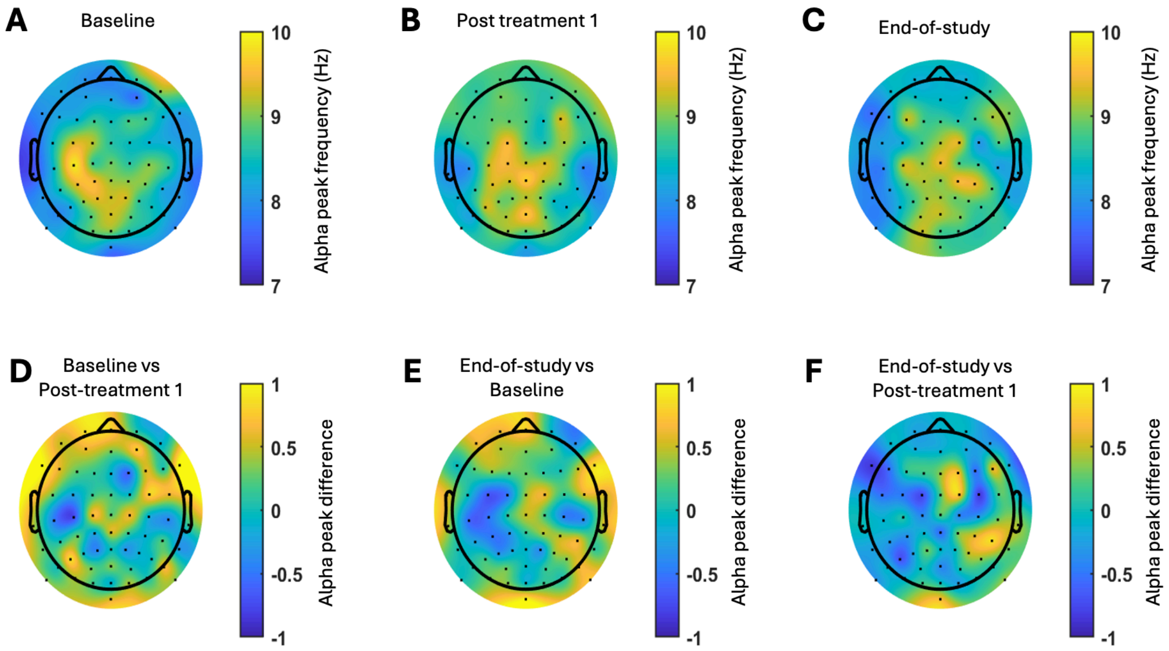

Supplementary Figure 2

EEG - Spectral power ratio. (A-D) Power (in μV^2) within oscillatory frequency bands Delta (A), Theta (B), Alpha (C) and Beta (D) and the averaged spectral power ratio ([alpha+beta]/[delta+theta] power) across the ROI (region of interest) for each session (E). Grey lines represent individual data. Black lines represent means. Error bars represent within-participants standard error of the mean. Spectral Power Ratios were compared between sessions with repeated-measures t-tests (N = 11 participants). One outlier participant was excluded due to data being outside the range of 3xIQR in all sessions). No comparisons were significant (all $t(11) > .74$, all $p > .443$. Post-treatment 1 = one day after SUS (scanning ultrasound) treatment 1, End-of-study = one day after SUS treatment 4.

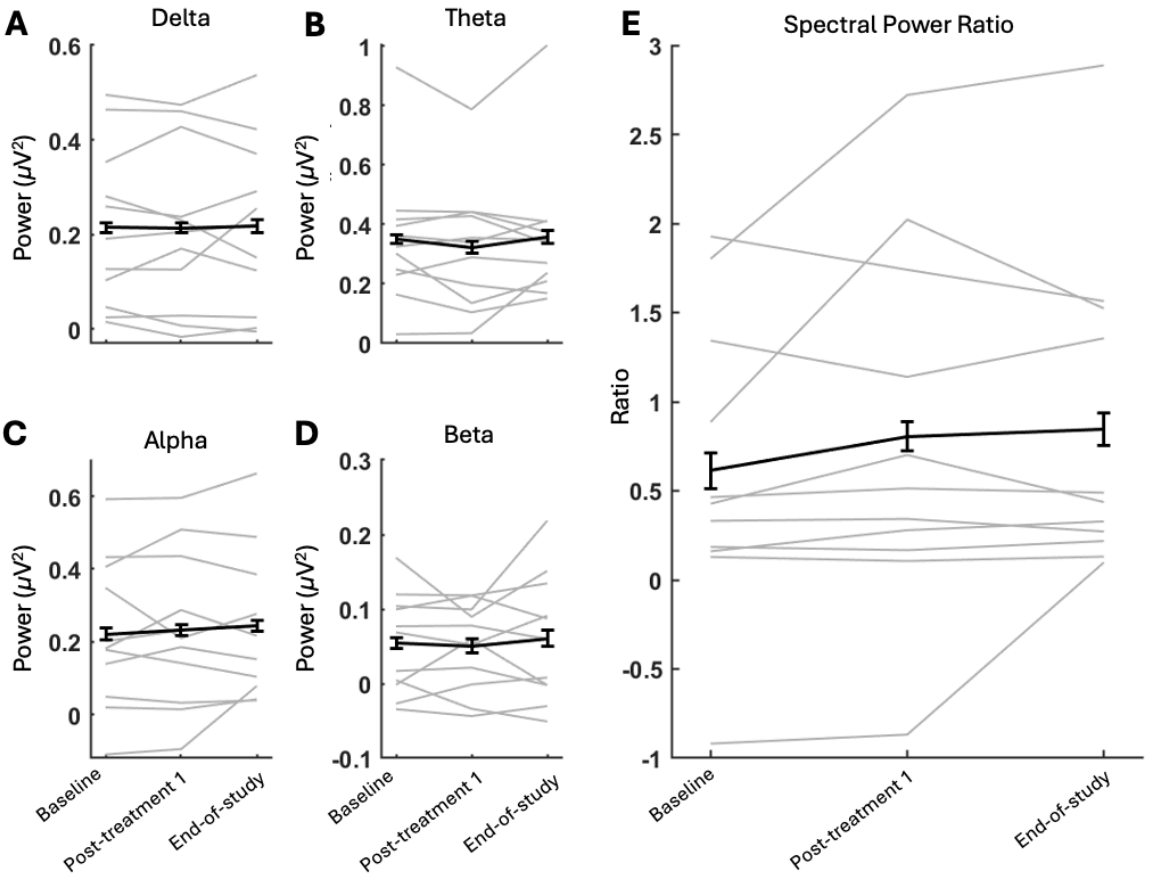

Supplementary Figure 3

Resting-state fMRI comparing functional activity and connectivity between pre- and post-treatment scans. (A) Comparison of the level of functional activity that changes between pre- and post-treatment scans in treatment, frontal control and default mode network (DMN) regions with individual data points shown for 11 study participants. One patient was excluded due to motion artifacts in the baseline MRI images. (B) Functional connectivity of treatment region and frontal control region between pre- and post-treatment scans. (C) Functional connectivity of treatment region and DMN between Baseline and End-of-study scans. The comparisons were performed using t-tests, on N=11 participants with t-values of ~ 0.5, 0.17, and 0.15 for sub-figures A, B, C respectively. Data points represent the value for each participant. In the plots, the violin shows the overall distribution shape (density estimate). The box and whiskers show key summary statistics: quartiles, median, and outlier boundaries (geom_violin() and geom_boxplot() functions in R).

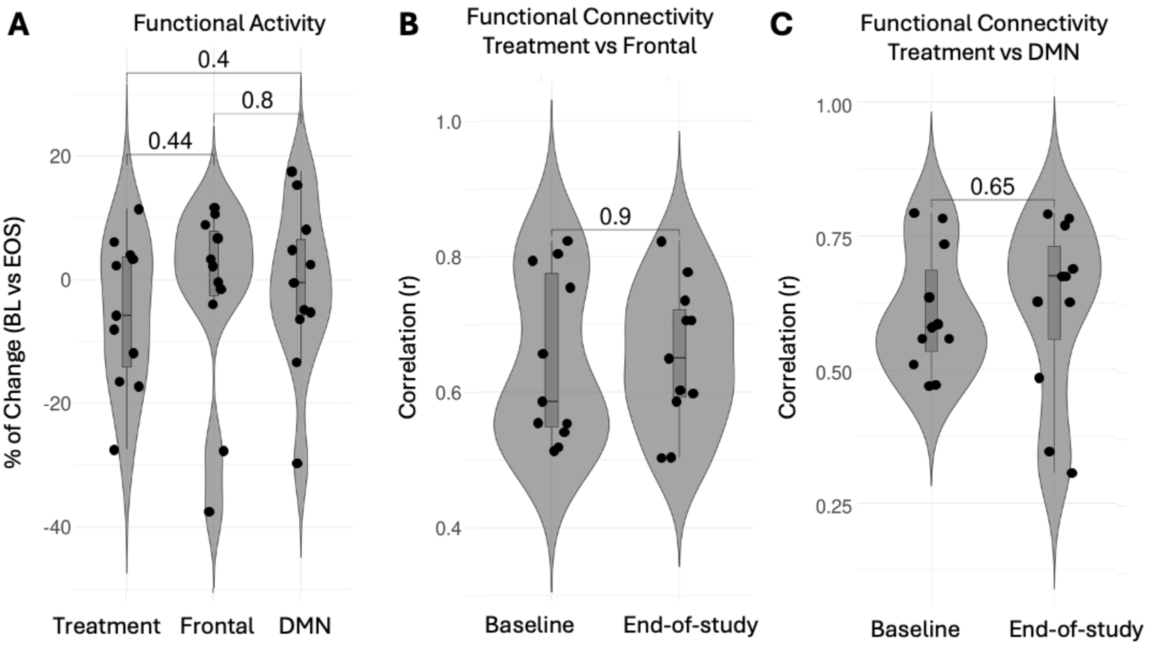

Supplementary Tables

Supplementary Table 1

Full inclusion and exclusion criteria.

**Inclusion criteria**
1. Confirmed AD: Clinical profile consistent with symptomatic AD plus a positive AD biomarker, at or prior to screening which could be either (i) positive amyloid PET scan (NAV4694 F18, florbetaben F18, florbetapir F18, or flutametamol F18), as read by the certified, site-designated PET scan reader or (ii) CSF biomarker profile analysis consistent with AD.
2. MMSE equal or > 10.
3. Age 50-85.
4. Clinically stable in the investigator's opinion.
5. If on anti-dementia drugs or other psychoactive medications (such as cholinesterase inhibitors, memantine or antidepressants) participants had to be on stable doses of drug for at least 2 months.
6. Participant's legally designated representative had to be able to freely give written informed consent; additionally, if, in the clinical judgment of the Principal Investigator, the participant themselves had the mental capacity to provide consent, they were also required to provide consent.
7. Ability to comply with study regimen and provide contact phone number.
8. Ability to communicate during procedures.
9. Presence of a regular caregiver (in daily contact with participant) who could attend appointments and provide information about adverse events.
10. Fully vaccinated for Covid-19, and willing to maintain vaccination status with booster vaccine if required.
**Exclusion criteria**
1. MRI contraindications:<br>a. Metallic objects in head, or presence of unknown or MR unsafe devices anywhere in body or,<br>b. Unable to tolerate MRI scanning (e.g. claustrophobia or known inability to lie sufficiently still from previous MRI scans).
2. Haemorrhages (including microhaemorrhages) on MRI scan.
3. Any other MRI findings that in the opinion of the study clinician may have been contributing to the clinical profile, including severe ischemic changes, active or chronic infection/inflammation, tumour/space occupying lesion, meningeal enhancement, intracranial hypotension.
4. Paget's disease of bone.
5. Clotting/bleeding disorder (including oral anti-coagulants; anti-platelet therapy such as low-dose aspirin was permitted).
6. Scalp or skull abnormalities.<br>a. Prior neurosurgical intervention of the brain / craniotomy or,<br>b. Skull area traversed by the sonication pathway was covered by scars, scalp disorders (e.g., eczema), open wounds, or atrophy of the scalp.
7. Other neurological diseases (or history of): severe traumatic brain injury, seizure disorders, stroke, tumours, transient ischemic attack.<br>a. Cerebral pathology unrelated to Alzheimer's disease.
8. History of major psychiatric disorders (such as major depression or schizophrenia).
9. History of drug or alcohol abuse.
10. An active inflammatory disease.
11. Active or recent history (within four weeks prior to screening) of a clinically significant bacterial, fungal, or mycobacterial infection.
12. Change of allowed, chronic, concomitant medication within 28 days prior to screening.
13. Pregnancy or breast-feeding.
14. Any other major medical illness that in the opinion of the study clinician could interfere with the intended use (e.g. unstable cardiovascular, pulmonary, hepatic or renal disease, active cancer etc.).
15. Any conditions that rendered the participant unable to lie flat for scanning.
16. Known cerebral or systemic vasculopathy.
17. Corticosteroid treatment within last 6 weeks before first treatment.

18. Increased intracranial pressure.
19. Known skin allergies or sensitivity to silicone, ultrasound gel or depilation cream.
20. Participation in other clinical trials within 90 days from the screening date.
21. An inability to communicate during a treatment procedure.
22. A body weight exceeding 200 kg.
23. Other conditions implying increased risk according to the judgement of the study clinician.

Supplementary Table 2

MRI parameters.

Sequence	Parameters	Matrix size	Total Acquisition Time
3-Dimensional Magnetization-Prepared Rapid Gradient-Echo Imaging (MPRAGE)	• 0.8 mm^3 spatial resolution<br>• repetition/echo/inversion times = 2,500/2.22/1,000 ms<br>• flip angle 8°<br>• field-of-view 208 × 208 × 188 mm<br>• bandwidth of 220 Hz/Px<br>• IPAT2 with reference line phase encoding (PE)=32	208 × 300 × 320	6:54 mins
T2-weighted isotropic turbo spin-echo (SPACE)	• 0.8 mm^3 spatial resolution<br>• repetition/echo times = 3200/563 *ms*<br>• field-of-view 208 × 208 × 188 mm<br>• bandwidth of 744 Hz/Px<br>• IPAT2 with reference line PE=32	208 × 300 × 320	5:57 mins
Multi-shell diffusion-weighted images (DWI)	• repetition/echo times = 4100/75*ms*<br>• Fractional anisotropy (FA) = 90°<br>• field-of-view 244 × 244 × 136 mm	122 × 122 × 68	7:00 mins
T2-weighted fluid-attenuated inversion recovery (FLAIR)	• 1.0 *mm* isotropic voxel<br>• repetition/echo/inversion times = 5000/388/1800 *ms*<br>• FA = 120°<br>• bandwidth of 751 Hz/Px<br>• IPAT2 with reference line PE =24	256 × 256 × 160	5:00 mins
Susceptibility-weighted images (SWI)	• 0.9 × 0.9 × 1.5 *mm* voxel<br>• TE/TR = 20.0/27.0 *ms*<br>• FA = 15°<br>• field-of-view 208 × 230 × 176 mm<br>• IPAT2 with PE=24<br>• bandwidth of 120 Hz/Px and flow compensation.	232 × 256 × 80	4:54 mins
T2*-weighted gradient-echo echo-planar imaging (EPI)	• TE/TR=30/735 *ms*<br>• FA = 52°,<br>• field of view = 208 × 208 × 160 mm	104 × 104 × 54, with a resolution of 2.0 × 2.0 *mm* and a slice thickness of 2 mm, with no gap between slices	6:09 mins

Supplementary Table 3

Summary of TEAEs by System Organ Class (SOC) and Preferred Term (PT).

System Organ Class (SOC)<br>Preferred Term (PT)	Cohort 1<br>(N=4)<br>n (%) E	Cohort 2<br>(N=8)<br>n (%) E	Overall<br>(N=12)<br>n (%) E
Subjects with at least one TEAE	2 (50.0) 5	1 (12.5) 3	3 (25.0) 8
Gastrointestinal disorders	-	1 (12.5) 2	1 (8.3) 2
Abdominal pain	-	1 (12.5) 1	1 (8.3) 1
Intestinal obstruction	-	1 (12.5) 1	1 (8.3) 1
Infections and infestations	1 (25.0) 1	-	1 (8.3) 1
COVID-19	1 (25.0) 1	-	1 (8.3) 1
Injury, poisoning and procedural complications	1 (25.0) 1	-	1 (8.3) 1
Arthropod bite	1 (25.0) 1	-	1 (8.3) 1
Nervous system disorders	2 (50.0) 2	-	2 (16.7) 2
Migraine	2 (50.0) 2	-	2 (16.7) 2
Skin and subcutaneous tissue disorders	1 (25.0) 1	1 (12.5) 1	2 (16.7) 2
Dermatitis	-	1 (12.5) 1	1 (8.3) 1
Pain of skin	1 (25.0) 1	-	1 (8.3) 1

Treatment Emergent Adverse Events (TEAEs) are defined as adverse events that occurred on or after the first study device treatment. If a subject has multiple occurrences of a TEAE, the subject is presented only once in the subject count (n) column, and occurrences are counted each time in the event (E) column. Percentages are based on population N. Medical Dictionary for Regulatory Activities (MedDRA) Version: 25.1

Supplementary references

1. Smith SM, Jenkinson M, Woolrich MW, Beckmann CF, Behrens TE, Johansen-Berg H, et al. Advances in functional and structural MR image analysis and implementation as FSL. NeuroImage. 2004; 23 Suppl 1: S208-19.
2. Oostenveld R, Praamstra P. The five percent electrode system for high-resolution EEG and ERP measurements. Clin Neurophysiol. 2001; 112: 713-9.
3. Donoghue T, Haller M, Peterson EJ, Varma P, Sebastian P, Gao R, et al. Parameterizing neural power spectra into periodic and aperiodic components. Nat Neurosci. 2020; 23: 1655-65.
4. Horvath A, Szucs A, Csukly G, Sakovics A, Stefanics G, Kamondi A. EEG and ERP biomarkers of Alzheimer's disease: a critical review. Front Biosci (Landmark Ed). 2018; 23: 183-220.
5. Kopcanova M, Tait L, Donoghue T, Stothart G, Smith L, Flores-Sandoval AA, et al. Resting-state EEG signatures of Alzheimer's disease are driven by periodic but not aperiodic changes. Neurobiol Dis. 2024; 190: 106380.
